# Supplementary figures and images for: Brain regional gene expression network analysis identifies unique interactions between chronic ethanol exposure and consumption
Source: PLoS One. 2020 May 29;15(5):e0233319. doi: 10.1371/journal.pone.0233319 (PMC7259766; doi:10.1371/journal.pone.0233319)

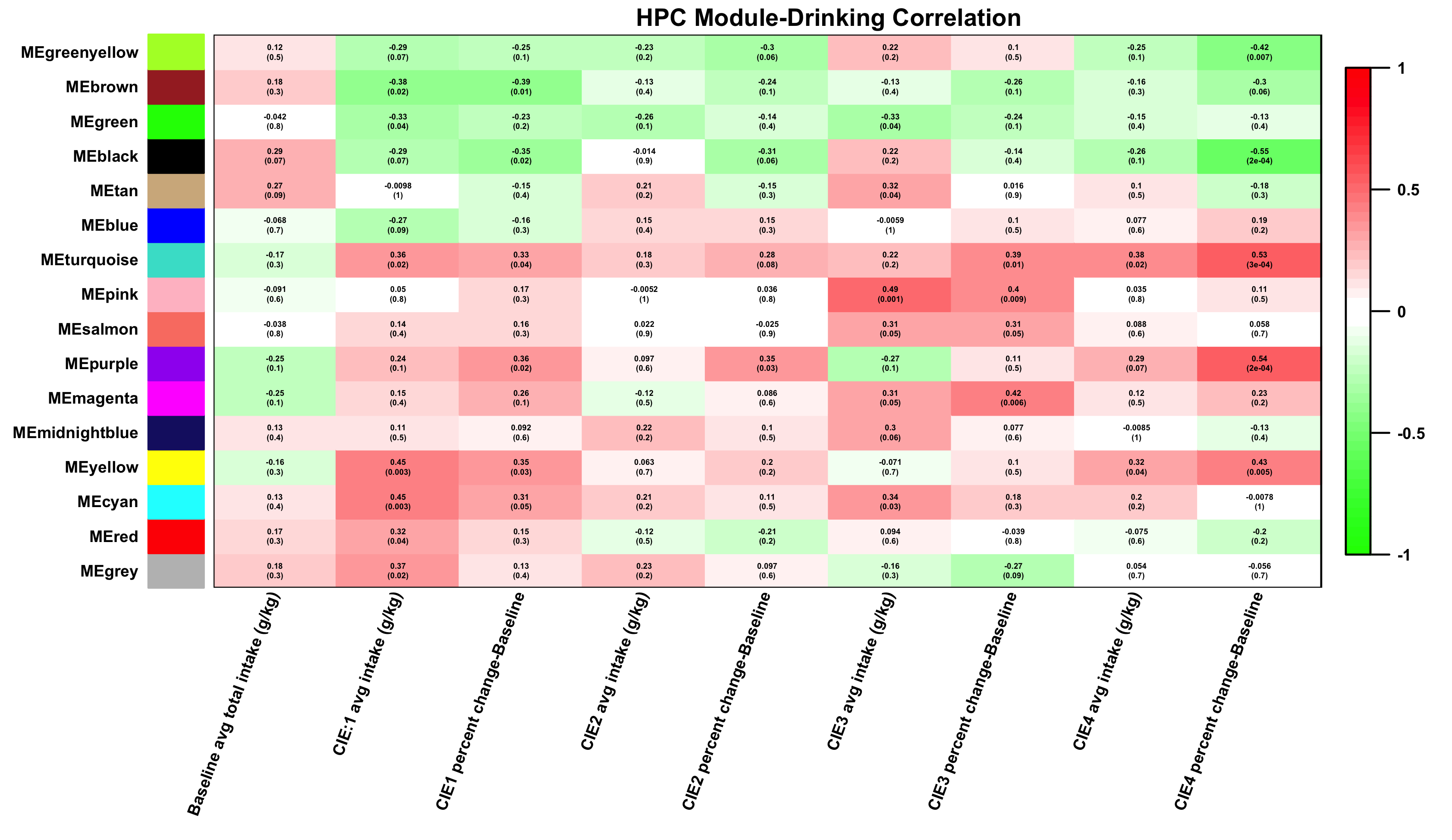

Supplement: S1 Fig — Eigengene values (1st principal component of gene expression) were correlated to ethanol intake measures. Cell color indicates strength of correlation (green = negative correlation, red = positive correlation). (TIFF) [file pone.0233319.s001.tiff]

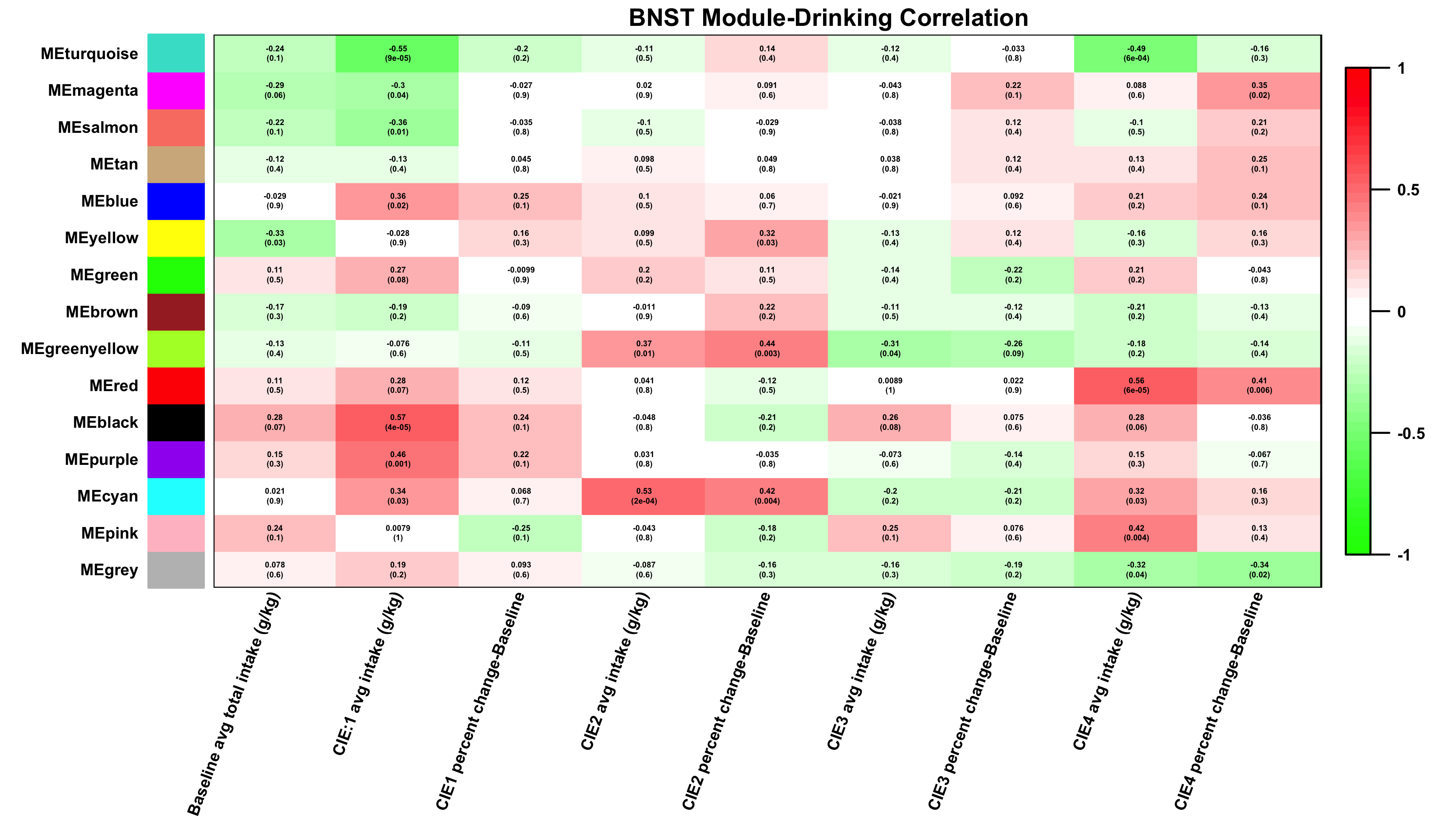

Supplement: S2 Fig — Eigengene values (1st principal component of gene expression) were correlated to ethanol intake measures. Cell color indicates strength of correlation (green = negative correlation, red = positive correlation). (TIFF) [file pone.0233319.s002.tiff]

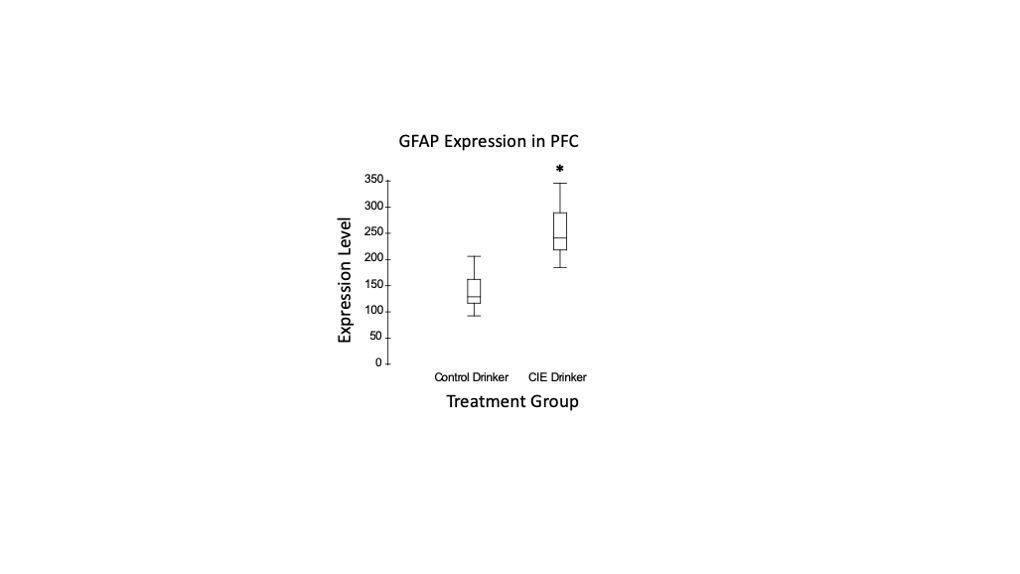

Supplement: S3 Fig — Comparison of Gfap expression (probeset 1440142_s_at) in Control Drinker vs. CIE Drinker groups in PFC. Y-axis reflects RMA expression values. *corrected p-value < 0.01. (TIFF) [file pone.0233319.s003.tiff]

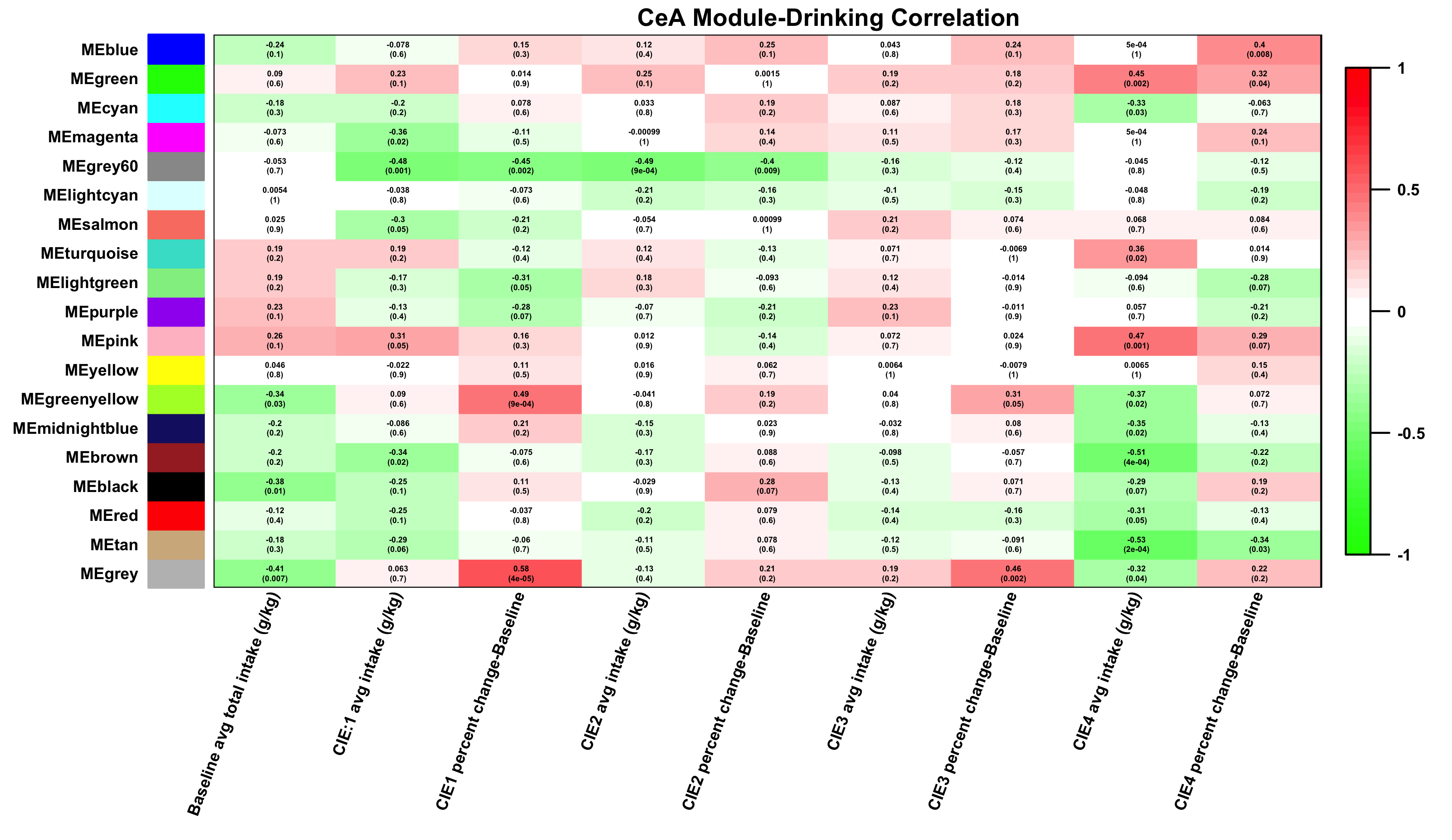

Supplement: S4 Fig — Eigengene values (1st principal component of gene expression) were correlated to ethanol intake measures. Cell color indicates strength of correlation (green = negative correlation, red = positive correlation). (TIFF) [file pone.0233319.s004.tiff]

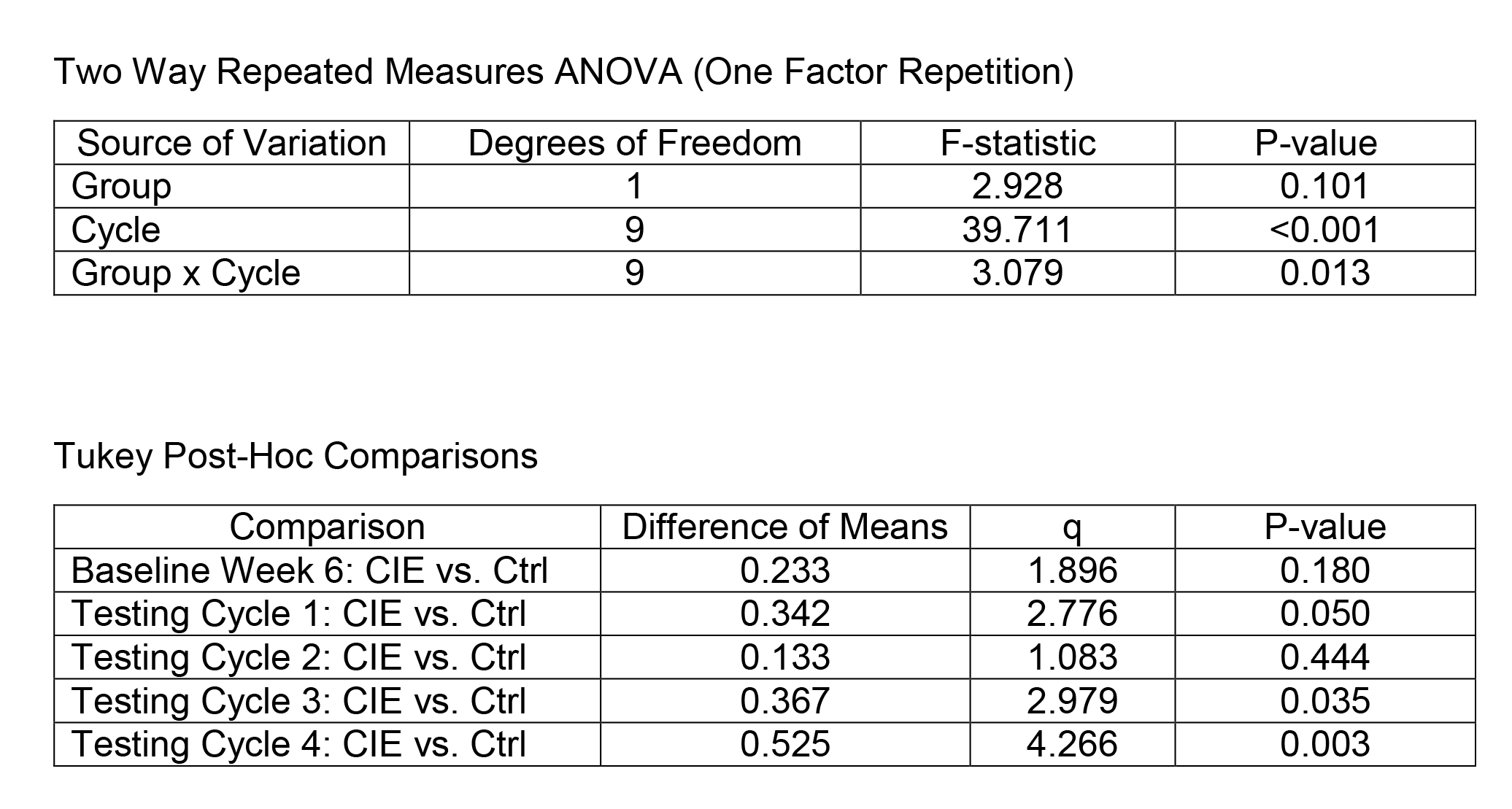

Supplement: S1 Table — Two-way repeated measures ANOVA comparing ethanol intake in g/kg between CIE and air control (ctrl). Significance: p-value ≤ 0.05. (TIFF) [file pone.0233319.s005.tiff]
